# MiR‐210‐3p‐EphrinA3‐PI3K/AKT axis regulates the progression of oral cancer

**DOI:** 10.1111/jcmm.15036

**Published:** 2020-03-17

**Authors:** Lin Wang, Yong Song, Hui Wang, Ke Liu, Zhe Shao, Zhengjun Shang

**Affiliations:** ^1^ The State Key Laboratory Breeding Base of Basic Science of Stomatology (Hubei‐MOST) & Key Laboratory of Oral Biomedicine Ministry of Education School & Hospital of Stomatology Wuhan University Wuhan China; ^2^ Department of Stomatology Liuzhou General Hospital Guangxi China

**Keywords:** cancer biology, cell signalling, epithelial‐mesenchymal interactions, gene expression, gene therapy/therapeutics, oral carcinogenesis

## Abstract

This study aimed to explore new therapeutic targets to improve the survival rate of patients with oral squamous cell carcinoma (OSCC).MiR‐210‐3p, EphrinA3 and EMT related indices were evaluated in OSCC tissues and cell lines. In addition, the relationship between differential EphrinA3 expression and tumour progression was explored through molecular biology techniques, in vitro functional experiments and tumour xenotransplantation models. The expression of EphrinA3 (*r_s_* = −0.719, *P* < .05) and E‐cadherin (*r_s_* = −0.856, *P* < .05) was negatively correlated with the pathological grading in OSCC tissues. Protein clustering shows EphrinA3 may be associated with tumour progression. EphrinA3 also can regulate the biological behaviour of oral cancer cells. And it regulates the EMT by the PI3K/AKT signalling pathway. MiR‐210‐3p targeted the gen EFNA3. Up‐regulation of miR‐210‐3p expression can decrease the expression of EphrinA3 and further to influence the biological behaviour of OSCC. The miR‐210‐3p‐EphrinA3‐PI3K/AKT signalling axis plays an important role in the progress of OSCC. EphrinA3 may serve as a novel target for oral cancer treatment.

## 1 INTRODUCTION

More than 90% of oral cancers are squamous cell carcinomas. Oral squamous cell carcinoma (OSCC) is a very aggressive neoplasm.^1^ OSCC progresses through the four stages of cancer and metastasizes to distant sites, including the buccal mucosa, the gingival, floor of mouth, palate and anterior tongue.^2^ Despite advances in cancer diagnosis and treatment, the overall 5‐year survival rate for OSCC remains the lowest among all malignancies.^3^ The epithelial‐mesenchymal transition (EMT) is a process wherein an epithelial cell loses its morphology and attains the morphological characteristics of a mesenchymal cell, which is important for the cancer metastasis.^4^, ^5^ In case of OSCC, the invasive front has been reported to express EMT markers, suggestive of the occurrence of this process during cancer progress.^6^ Hence, EMT has a prognostic significance in OSCC. The induction of EMT in OSCC has been attributed to multiple oncogenic pathways, including the phosphatidylinositol‐4,5‐bisphosphate 3‐kinase (PI3K)/protein kinase B (AKT) signalling pathway, transforming growth factor‐β (TGF‐β)/Smad pathway, Wnt pathway and Notch pathway.^7^

Ephrins and their receptors Eph may transmit bidirectional signals to regulate various developmental and normal physiological processes in human.^8^ EphA receptors include EphA1‐A8, while ephrinA ligands include ephrinA1‐A5. EphA receptors usually only bind to ephrinA ligands in a non‐specific manner.^9^, ^10^ Moreover, the inhibition of ephrin in turn causes inhibition of ligand‐dependent migration and invasion of tumour cells.^11^ Our previous research revealed the overexpression of EphA2 and ephrinA1 in OSCC and salivary adenoid cystic carcinoma.^12^, ^13^, ^14^ EphrinA3 plays an important role in the growth and development of the embryonic nervous system, but its role in the occurrence and progression of tumours is unclear.

The reported target genes of miR‐210‐3p include *HOXA1*, *HOXA9*, *HOXA3*, *E3F3* and EphrinA3 (*EFNA3*);^15^ however, only *EFNA3* was shown to be regulated by miR‐210‐3p under hypoxic conditions.^16^

We knew that hypoxia was associated with tumour progression and previous studies have linked EFNA3 to hypoxia.^15^ Since hypoxia plays an important role in the occurrence and development of oral squamous cell carcinoma (OSCC), we wanted to discuss the role of EphrinA3 in the development of OSCC.

## 2 MATERIALS AND METHODS

### 2.1 Patients and samples

Human OSCC primary samples (n = 53) were collected at the Hospital of Stomatology, Wuhan University, from 2013 to 2015. The study was approved by the Wuhan university ethics committee. All patients provided written informed consent. All patients underwent potentially curative surgery without preoperative therapy. Histologic specimens from each patient were reviewed to confirm the diagnosis of squamous cell carcinoma. Twenty normal mucosa tissues were chosen as controls. Clinical staging was performed according to the 2018 criteria of the International Union Against Cancer. This study was approved by the Ethics Committee of Hospital of Stomatology, Wuhan University, and informed consent was obtained from all patients.

### 2.2 Antibodies and immunohistochemistry

Immunohistochemical studies were performed using the following antibodies: EphrinA3‐specific polyclonal antibody (dilution 1:100) from Santa Cruz Biotechnology Inc (Santa Cruz, CA); anti‐E‐cadherin antibody (1:1000, CST); SP immunochemical test kit purchased from MaiXin Ltd. (FU Zhou, China). Microarrays were prepared from 53 oral cancer tissues and 20 normal oral mucosa tissues. Slides of 3‐μm thickness serial sections of the tissue microarray were prepared. EphrinA3 and E‐cadherin staining was assessed according to a score that added a scale of intensity of staining (magnification ×200) to the proportion of stained cells (magnification ×40), as previously described.^17^ The average optical density (AOD) value of EphrinA3 and E‐cadherin staining was calculated using a semiautomated computerized image analysis system (Image‐Pro Plus 6.0; Media Cybernetics, Bethesda, USA). For each section, the average AOD score was calculated from triplicate values. And the average AOD represented the expression of EphrinA3 and E‐Cadherin.

### 2.3 In situ hybridization histochemistry

In situ hybridization histochemistry (ISHH) was performed with Has‐miR‐210‐3p probe: TCAGCCGCTGTCACACGCACAG. The mRNA ISH Kit was purchased from BosterBio (USA). The samples were treated with 0.1 M glycine‐deionized aldehyde group for 15 minutes at 37°C, followed by treatment with proteinase K for 30 minutes. The slides were incubated with an alkaline phosphate‐labelled antibody (1:500) diluted with an antibody diluent at 37°C, followed by treatment with anti‐digoxin for 3 hours. The tissue microarray was finally dewatered and sealed.^18^

### 2.4 Cell lines and culture

Human OSCC lines, Cal‐27 and SCC‐25 kindly donated by Professor Zhuan‐Bian were purchased from the American Type Culture Collection (ATCC, Manassas, VA, US). OSCC lines were cultured in Dulbecco's modified Eagle's medium (DMEM) high‐glucose (HyClone, UT, USA) supplemented with 10% foetal bovine serum (FBS; Gibco, Carlsbad, Calif, USA). Human immortalized oral epithelial cells (HIOECs) were kindly provided by Professor Cheng‐zhang Li and Doctor Zhen‐Zhang and were cultured in KGM gold (Lonza, Walkersville, MD) supplemented with 5% FBS and KGM gold growth factor mixture. All the control cells were cultured in an incubator with 5% CO_2_ at 37°C.

### 2.5 Cell transduction and screening of stable cells

The recombination lentiviral vectors of EphrinA3‐RNAi were purchased from GenePharma, Shanghai, China. The cultured Cal‐27 and SCC‐25 cells were added to lentiviral supernatants containing EphrinA3‐RNAi vectors. After transduction for 72 hours, Cal‐27 and SCC‐25 cells carrying EphrinA3‐RNAi were selected with 10 μg/mL of puromycin. The efficiency of transduction was evaluated with immunofluorescence (Carl Zeiss, Germany).

### 2.6 Small‐interfering RNA transfection

Cal‐27 and SCC‐25 cells were transfected with EphrinA3 Small‐interfering RNA (siRNA) (synthesized by Gene Pharma Co., Shanghai, China) using lipofectamine 2000 transfection reagent (Invitrogen). The sequences targeting *EFNA3* were 5′‐TAGGAGGCCAAGAACGTCATG‐3′ (sense) and 5′‐ATCCTCCGGTTCTTGCAGT‐3′ (anti‐sense), and the sequences of the scramble siRNA were 5′‐TTCTCCGAACGTGTCACGT‐3′ (sense) and 5′‐AAGAGGCTTGCACAGTGCA‐3′ (anti‐sense). After 48 hours, the proteins were harvested to confirm the down‐regulation of EphrinA3 expression, and the cells were collected for further analysis.

### 2.7 Protein extraction and Western blot analysis

Total protein of Cal‐27 and SCC‐25 cells was extracted using M‐PER (Pierce Inc, USA) supplemented with protease inhibitor and phosphatase inhibitor on ice. The protein bands were transferred onto polyvinylidene difluoride (PVDF) membranes in a transfer buffer for 2 hours at 200 mA. The membranes were incubated with anti‐glyceraldehyde‐3‐phosphate dehydrogenase (GAPDH) antibody (1:10 000) (Proteintech, Wuhan, China), anti‐EphrinA3 antibody (1:400, Santa Cruz, CA), anti‐E‐cadherin antibody (1:1000, CST), anti‐N‐cadherin antibody (1:1000, CST), anti‐AKT antibody (1:1000, CST) and anti‐p‐AKT (Ser473) antibody (1:2000, CST) overnight at 4°C. The bound antibodies were tested with horseradish peroxidase‐conjugated anti‐rabbit IgG or anti‐mouse IgG (Pierce Chemical, Rockford, IL, USA).

### 2.8 Real‐time reverse‐transcriptase (RT) polymerase chain reaction (PCR)

Total RNA was extracted from Cal‐27 and SCC‐25‐vector (it represented SCC25 and Cal‐27 cell lines which transfected with empty vector virus as blank control group) and Cal‐27 and SCC‐25‐EphrinA3‐RNAi cells (it represented SCC25 and Cal‐27 cell lines constructed after the virus which can knockdown the EFNA3 gene was transferred) and Cal‐27 and SCC‐25‐EphrinA3‐mimics (it represented the SCC25 and Cal‐27 cell lines which overexpressed the protein EphrinA3) using TRIzol reagent (Invitrogen, Carlsbad, USA). Gene‐specific primers employed for cDNA amplification were synthesized as follows: EphrinA3 forward, 5′‐GACCTTCTGGCACATACTAACTACACC‐3′ and reverse, 5′‐CAGGCTTGAGGCTACTGATGGTAAC‐3′; E‐cadherin forward, 5′‐AGTCACTGACACCAACGATAAT‐3′ and reverse, 5′‐ATCGTTGTTCACTGGATTTGTG‐3′; N‐cadherin forward, 5′‐CGATAAGGATCAACCCCATACA‐3′ and reverse, 5′‐TTCAAAGTCGATTGGTTTGACC‐3′; β‐actin forward, 5′‐CATTAAGGAGAAGCTGTGCT‐3′ and reverse, 5′‐GTTGAAGGTAGTTTCGTGGA‐3′ (Sangon Biotech, Shanghai, China). Relative levels of EphrinA3, E‐cadherin and N‐cadherin were determined after normalization with an endogenous control β‐actin and calculated from the standard curve. All real‐time RT‐PCR tests were performed in triplicates. The comparative Ct method was used for the final results.

### 2.9 Cell proliferation assay

Cells were seeded in 96‐well culture plates, and cell proliferation was determined after incubation for 24 and 48 hours using a cell counting kit‐8 (CCK8; Dojindo, Japan). Growth curves were generated from a colorimetric assay. The absorbance value of each well at 450 nm was measured with a micro‐plate reader (Thermo MutliscanMK3; Thermo Fisher Scientific, Waltham, USA).

### 2.10 Migration and invasion assay

Invasion and protein‐nucleic acid interaction (PNI) assays were performed using transwell chambers (Corning, Tewksbury, USA) with a polycarbonate membrane (6.5 mm diameter, 8 μm pore size). The membrane was coated with 50 μL of 8 mg/mL reconstructed extracellular matrix (Matrigel; BD Biosciences, USA) prior to use. In the lower chamber, 600 μL of conditioned medium was used as a chemoattractant. Exponentially growing cells were harvested and seeded into the upper chambers at 2 × 10^5^ cells/well in DMEM supplemented with 1% FBS. Cells were incubated in a humidified atmosphere at 37°C with 5% CO_2_ for 10 hours. Invaded cells were fixed, stained and counted under a light microscope at a magnification of 400×.

### 2.11 Tumour xenograft model

All animal experiments were approved by the animal ethics committee of wuhan university, and the animals were executed without suffering. Sixteen BALB/C nude mice purchased from SLAC Laboratory Animal Centre (Hunan, China) were fed under specific disease‐free (SPF) conditions and randomly divided into four groups. Approximately 200 μL of single cell suspension (2 × 10^6^ cells) inoculated into the right axillary fossa of mice from the four groups. Six weeks after inoculation, tumour tissues were removed and subjected to immunohistochemistry assay.

### 2.12 Proteomic analysis

The proteins were determined through reverse‐phase high‐pressure mass spectrometry via NanoLC‐Ultra 1D (Eksigent) and TripleTOF 5600+ (AB Sciex) as previously reported.^19^ The raw EBI data were searched using the ProteinPilot software. To sort through the large number of regulated proteins, the Gene Ontology (GO) pathway database was accessed via the GO automatic annotation server (ANNEX).

### 2.13 Statistical analysis

Categorical variables were expressed as percentage and analysed with Fisher's exact test. The cut‐off point to convert a continuous variable into a categorical value was calculated with a lower p value.^19^ Parameters considered statistically significant (*P* < .10) in the univariate model were analysed in the multivariate models. All two‐sided *P* values < 0.05 were considered as significant. All analyses were carried out using SPSS software (SPSS V.17.0, Chicago, Illinois, USA).

## 3 RESULTS

### 3.1 Correlation between miR‐210‐3p, EphrinA3, E‐cadherin and tumour pathological stage in OSCC

**Figure 1:**
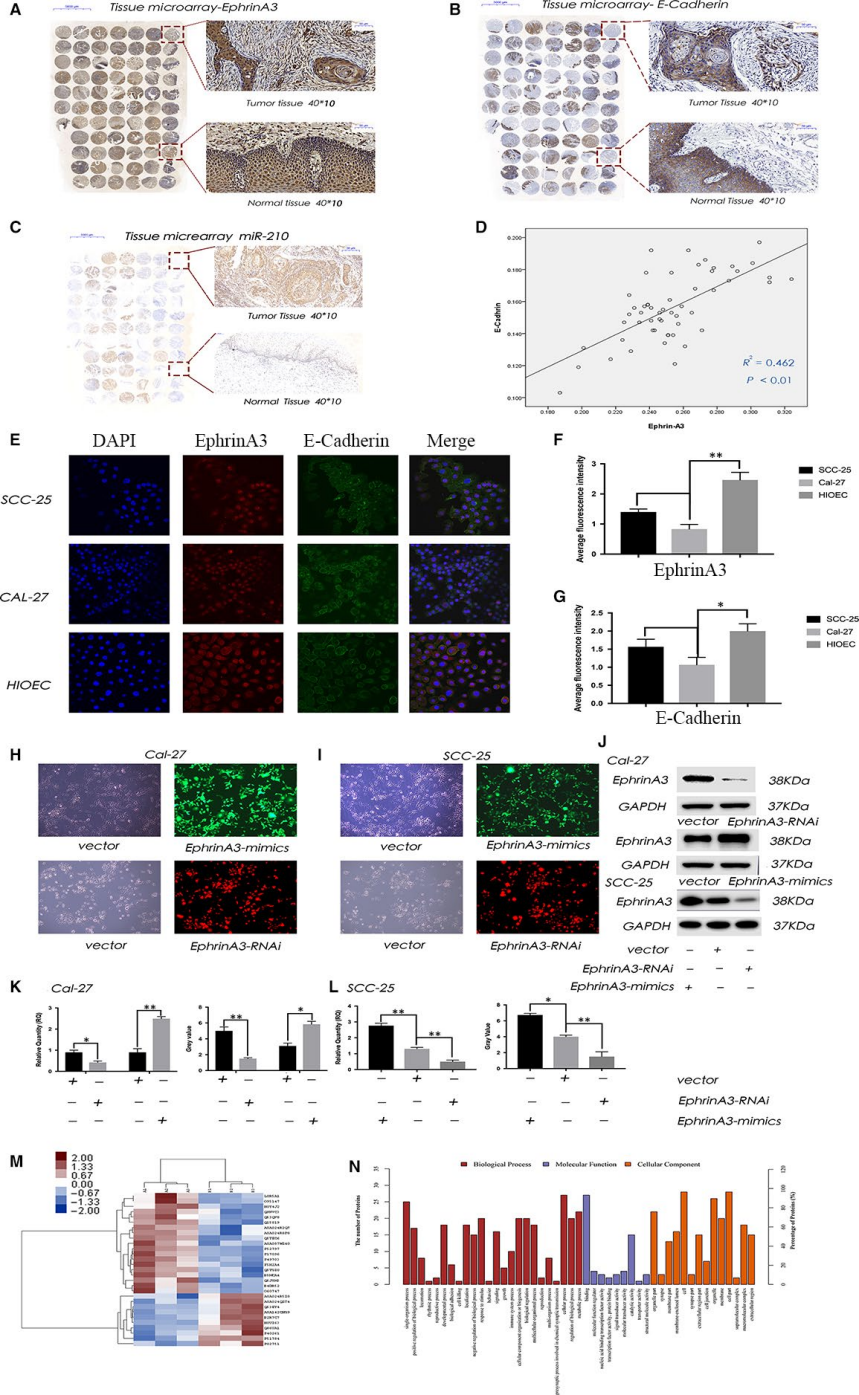
Correlation between miR‐210‐3p, EphrinA3, E‐cadherin and tumour pathological stage in OSCC. A‐C, show that miR‐210, EphrinA3 E‐Cadherin are expressed in human oral squamous cell carcinoma tissue specimens. D, the average optical density (AOD) of EphrinA3 and E‐cadherin in 53 cases of oral squamous cell carcinoma (OSCC) satisfied Normal Distribution, and there was a correlation between them (*R* ^2^ = .462, *P* < .01). E, shows that EphrinA3 and E‐cadherin were expressed in both OSCC cells and immortalized oral mucosal epithelial cell lines (HIOEC). F, G, showed the expression of EphrinA3 and E‐Cadherin in OSCC cells and HIOEC. The expression in HIOEC was also significantly higher than that in OSCC cells (*P* < .05). H, I, showed Cal‐27 and SCC‐25 cell lines were transfected the EphrinA3‐RNAi and the EphrinA3‐mimics. J‐L, respectively verified the successful transfection modelling from RNA and protein levels. M, shows the results of protein clustering analysis. Overexpressed EphrinA3 and vector differentially expressed proteins. The darker the colour, the greater the difference. N, classifies these differentially expressed proteins from three aspects: the biological processes involved in the proteins, the molecular functions they have and the cell components they are in

EphrinA3 was a direct target of miR‐210‐3p. Modulation of EphrinA3 expression by miR‐210‐3p had significant functional consequences.^20^ The results revealed the expression of miR‐210‐3p, EphrinA3, and E‐cadherin in OSCCs and normal mucosa tissues (Figure 1A‐C). Image‐Pro Plus 6.0 was used to analyse the AOD of EphrinA3 and E‐cadherin in 53 cases of OSCCs and revealed a correlation between them (*R* ^2^ = .462, *P* < .01) (Figure 1D). EphrinA3 expression negatively correlated with miR‐210‐3p. A multifactorial study on the pathological grading of 53 OSCC tissues using rank test showed that EphrinA3 (*r_s_* = −.719, *P* < .05) and E‐cadherin (*r_s_* = −.856, *P* < .05) had negative correlations with pathological grading (Table 1). The EphrinA3 expression decreased in the high‐grade tumour. Kruskal‐Wallis one‐way analysis of variance (ANOVA) was carried out to compare the EphrinA3 expression with different pathological grade: (a) grade I versus II (*P *< .01); (b) grade I versus III (*P < *.01); (c) grade II versus III (*P* = .357) (Table 2). And we also measured the expression of EphrinA3 and E‐cadherin in oral squamous cell lines (SCC‐25 and Cal‐27) and immortalized oral mucosal epithelial cell lines (HIOEC) (Figure 1E) by using the average AOD. The expression of EphrinA3 and E‐Cadherin in HIOEC was also significantly higher than that in OSCC cells (*P* < .05) (Figure 1F and G).

**Table 1:** Analysis of correlation factors of pathological grade

	The pathological grade	
	*r_s_*	*P*
EphrinA3	−.719	.003^*^
E‐Cadherin	−.856	.033^*^

Note

In oral cancer tissue specimens, ephrinA3 was negatively correlated with pathological grade (*r_s_* = −.719, *P < .05); E‐Cadherin was also negatively correlated with pathological grade (*r_s_* = −0.856, *P < .05).

**Table 2:** Multivariate analysis of EphrinA3 and tumour clinical staging

Variables	Years (Mean ± SD)	n	EphrinA3 (M,Q)	H	Z	*P*
Gender						
Male		38				
Female		15				
Age	57.51 ± 12.88					
Pathological grade						
I		15	7.47,2.58	27.48		<.01*
II		33	4.33,0.89			
III		5	2.65,2.34			
Clinical grade						
I		4	4.75,1.77	2.21		.529
II		14	4.82,3.28			
III		23	4.31,1.30			
IV		12	5.21,2.45			
T						
1‐2		34	4.82,2.70		‐1.345	.179
3‐4		19	4.27,2.09			
N						
0		26	4.82,3.22		‐1.094	.274
1‐3		27	4.56,1.68			

Note

Kruskal‐Wallis one‐way (ANOVA) was carried out between the following two groups: (1) pathological grade I group versus pathological grade II group: *P < .01; (2) pathological grade I group versus pathological grade III group: *P < .01; (3) pathological grade II group versus pathological grade III group: *P = .357.

### 3.2 Protein clustering shows EphrinA3 may be associated with tumour progression

Firstly, we transfected Cal‐27 and SCC‐25 cell lines with the EphrinA3‐RNAi and the EphrinA3‐mimics (Figure 1H and I). And we also verified the successful transfection modelling from RNA and protein levels (Figure 1J‐L). The total protein from Cal‐27 cells overexpressing EphrinA3 and vector group were extracted, and the proteomics were used to quantify the differentially expressed proteins. The differentially expressed proteins were named using the *Homo sapiens* database.^21^ The database search was conducted using MASCOT 2.6.^22^ Isobaric tags for relative and absolute quantitation (iTRAQ) were used to quantify the differentially expressed proteins. A total of 24 differentially expressed proteins met the screening criteria. Of these, 17 were up‐regulated and 7 showed down‐regulated expression. The darker the colour, the greater the difference (Figure 1M). The enrichment analysis of GO annotation is used to evaluate the significance level of a certain GO term protein enrichment by Fisher's exact test.^23^ And it classified these differentially expressed proteins from three aspects: the biological processes involved in the proteins, the molecular functions they have and the cell components they are in (Figure 1N).

### 3.3 EphrinA3 can regulate the biological behaviour of oral cancer cells

**Figure 2:**
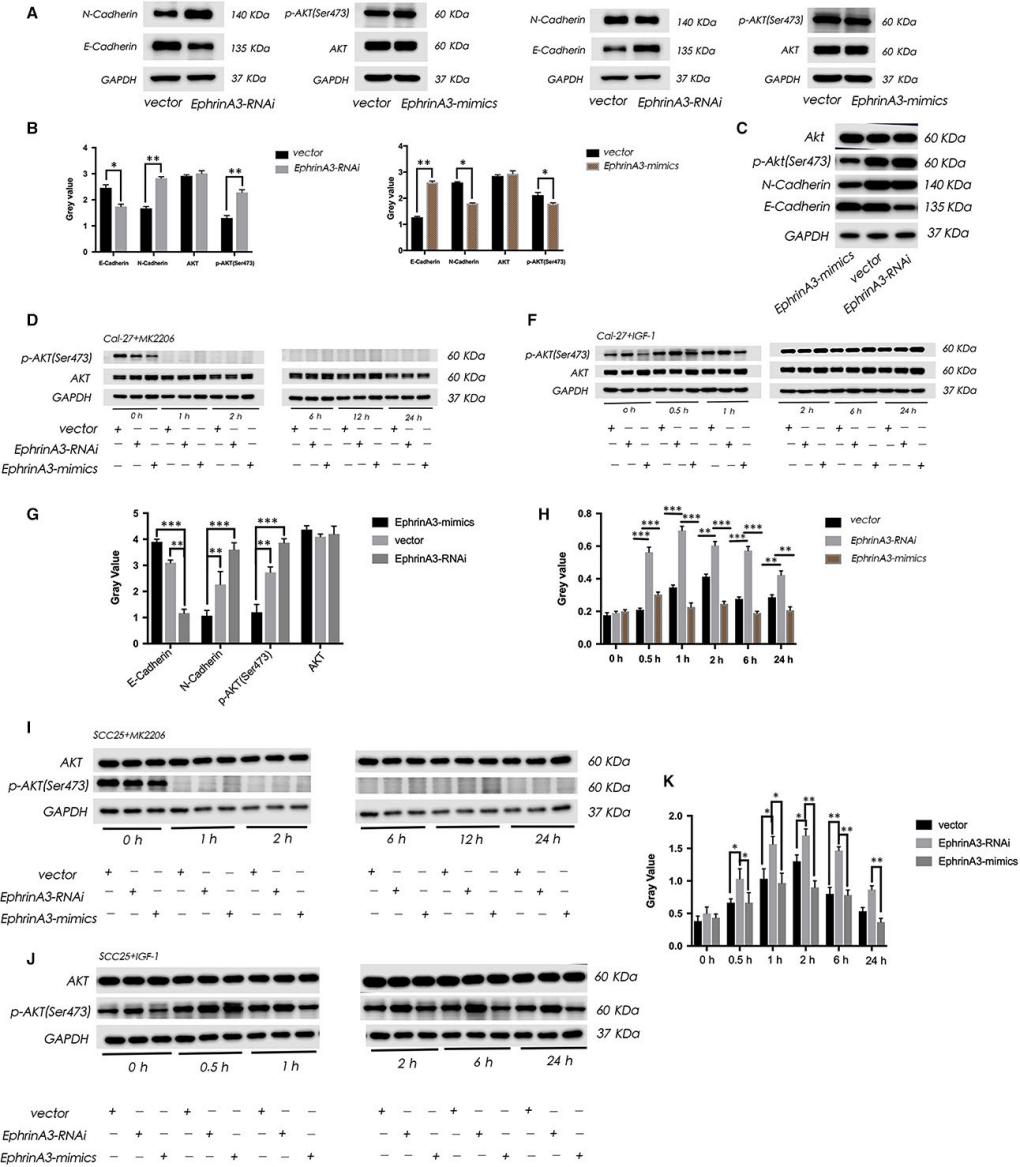
EphrinA3 regulates the epithelial‐mesenchymal transition (EMT) by the PI3K/AKT signalling pathway. A‐C, showed in Cal‐27 and SCC‐25 cell lines which transfected with EphrinA3‐RNAi, the decrease of EphrinA3 expression caused the decrease of E‐Cadherin expression, while the expression of N‐cadherin increased. And the expression of p‐AKT (Ser473) increased which indicated that the PI3K/AKT pathway activated. But when transfected with EphrinA3‐mimics, the overexpression of EphrinA3 caused the overexpression of E‐Cadherin, but the expression of N‐cadherin decreased. And the expression of p‐AKT reduced which indicated that the PI3K/AKT pathway inhibited. D‐K, showed that when added the inhibitor (MK2206) of the PI3K/AKT pathway, the expression of p‐AKT reduced in group vector, EphrinA3‐RNAi, and EphrinA3‐mimics. But when added the agonist (IGF‐1), we found that the expression of *p*‐AKT increased in three groups. And the level of *p*‐AKT in EphrinA3‐RNAi group was higher than that in vector group. The level of *p*‐AKT in EphrinA3‐mimics group was generally lower than that in vector group (*P* < .05)

**Figure 3:**
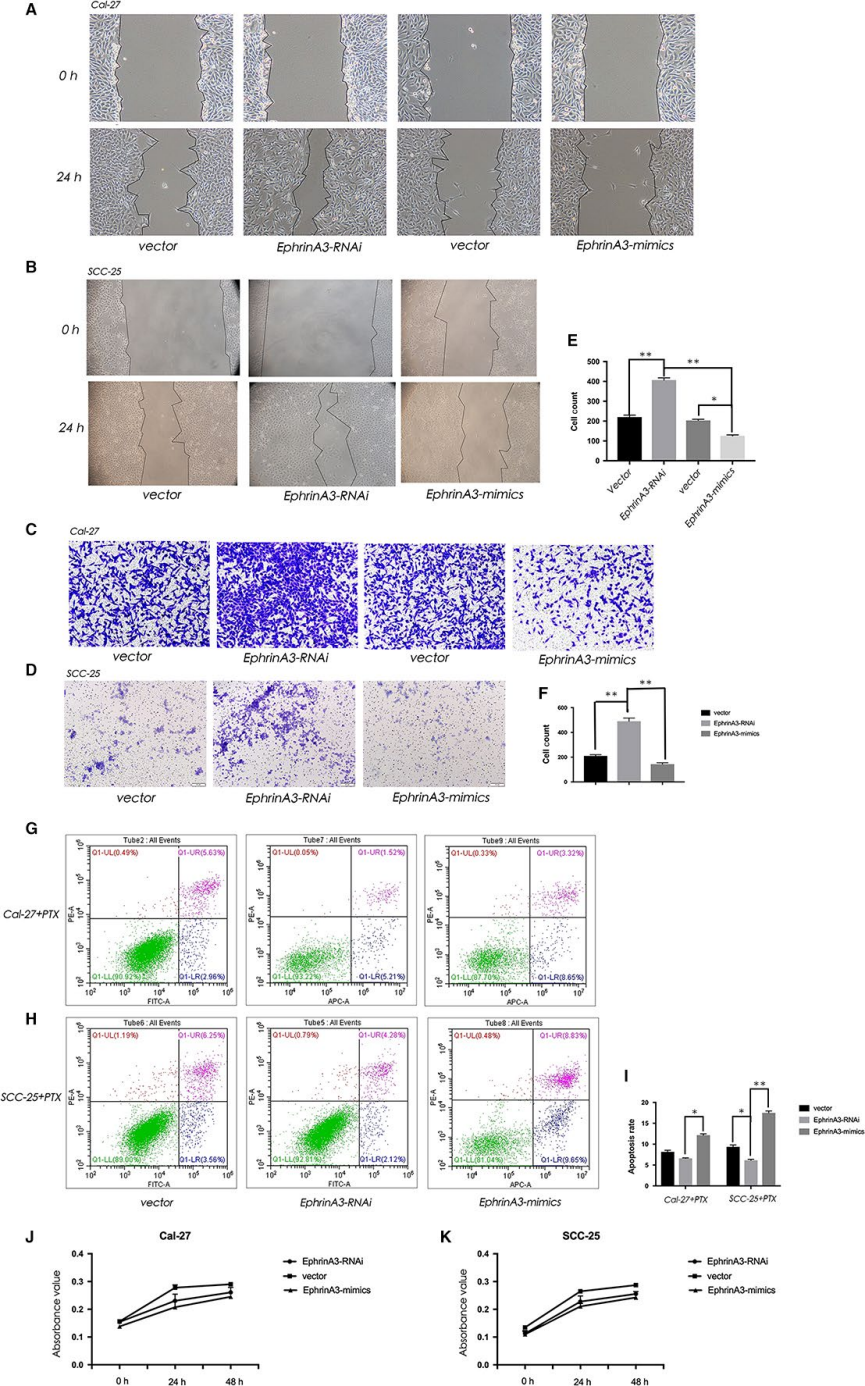
EphrinA3 can regulates the biological behaviour of oral cancer cells. A, B, showed at 24 h, the scratch healing ability of Cal‐27 and SCC‐25 cells in the EphrinA3‐RNAi group was higher than that in EphrinA3‐mimics group. C‐F, showed that at 12 h, the invasion ability of Cal‐27 and SCC‐25 cells in EphrinA3 knockdown group was higher than that in EphrinA3 overexpression group and the difference was statistically significant (*P* < .05). G‐I, showed the drug resistance in three groups. When treated with PTX, The apoptosis rate of EphrinA3‐RNAi group was lower than that of vector group and that of EphrinA3‐mimics group was higher than that of vector group. And the difference was statistically significant (*P* < .05). G, K, the proliferation ability of Cal‐27 and SCC‐25 cells in the EphrinA3 knockdown group was stronger than that in the EphrinA3 overexpression group

Up‐regulation or down‐regulation of EphrinA3 could lead to changes of biological behaviour of OSCC cells. At 24 hours, the scratch healing ability of Cal‐27 and SCC‐25 cells in the EphrinA3‐RNAi group (it represented SCC25 and Cal‐27 cell lines constructed after the virus which can knockdown the EFNA3 gene was transferred) was stronger than that in EphrinA3‐mimics group (it represented the SCC25 and Cal‐27 cell lines which overexpressed the protein EphrinA3) (Figure 3A, B). At 12 hours, the invasion ability of Cal‐27 and SCC‐25 cells in EphrinA3 knockdown group was stronger than that in EphrinA3 overexpression group and the difference was statistically significant (*P* < .05) (Figure 2 C‐F). It also influenced the drug resistance. When treated with PTX（paclitaxel）, the apoptosis rate of EphrinA3‐RNAi group was lower than that of vector group (it represented SCC25 and Cal‐27 cell lines which transfected with empty vector virus as blank control group) and that of EphrinA3‐mimics group was higher than that of vector group. And the difference was statistically significant (*P* < .05; Figure 2J‐I). And the proliferation ability of Cal‐27 and SCC‐25 cells in the EphrinA3 knockdown group was stronger than that in the EphrinA3 overexpression group (Figure 2J, K).

### 3.4 EphrinA3 regulates the epithelial‐mesenchymal transition by the PI3K/AKT Signalling pathway

In Cal‐27 and SCC‐25 cell lines which were transfected with EphrinA3‐RNAi, the decrease of EphrinA3 expression caused the decrease of E‐Cadherin expression, while the expression of N‐cadherin increased. And the expression of p‐AKT (Ser473) increased which indicated that the PI3K/AKT pathway activated. But when transfected with EphrinA3‐mimics, causing the expression of E‐Cadherin increased, but the expression of N‐cadherin decreased. And the expression of p‐AKT reduced which indicated that the PI3K/AKT pathway inhibited (Figure 3A‐C). When we further added the inhibitor (MK2206) of the PI3K/AKT pathway, the expression of p‐AKT reduced in group vector, EphrinA3‐RNAi and EphrinA3‐mimics. But when added the agonist (IGF‐1), the expression of *p*‐AKT increased in three groups. And the level of *p*‐AKT in EphrinA3‐RNAi group was higher than that in vector group. But the level of *p*‐AKT in EphrinA3‐mimics group was lower than that in vector group (*P* < .05) (Figure 3D‐K). So we inferenced that the EphrinA3 regulates the epithelial‐mesenchymal transition (EMT) by the PI3K/AKT signalling pathway.

### 3.5 Suppression of EphrinA3 expression promotes tumour proliferation in vivo

**Figure 4:**
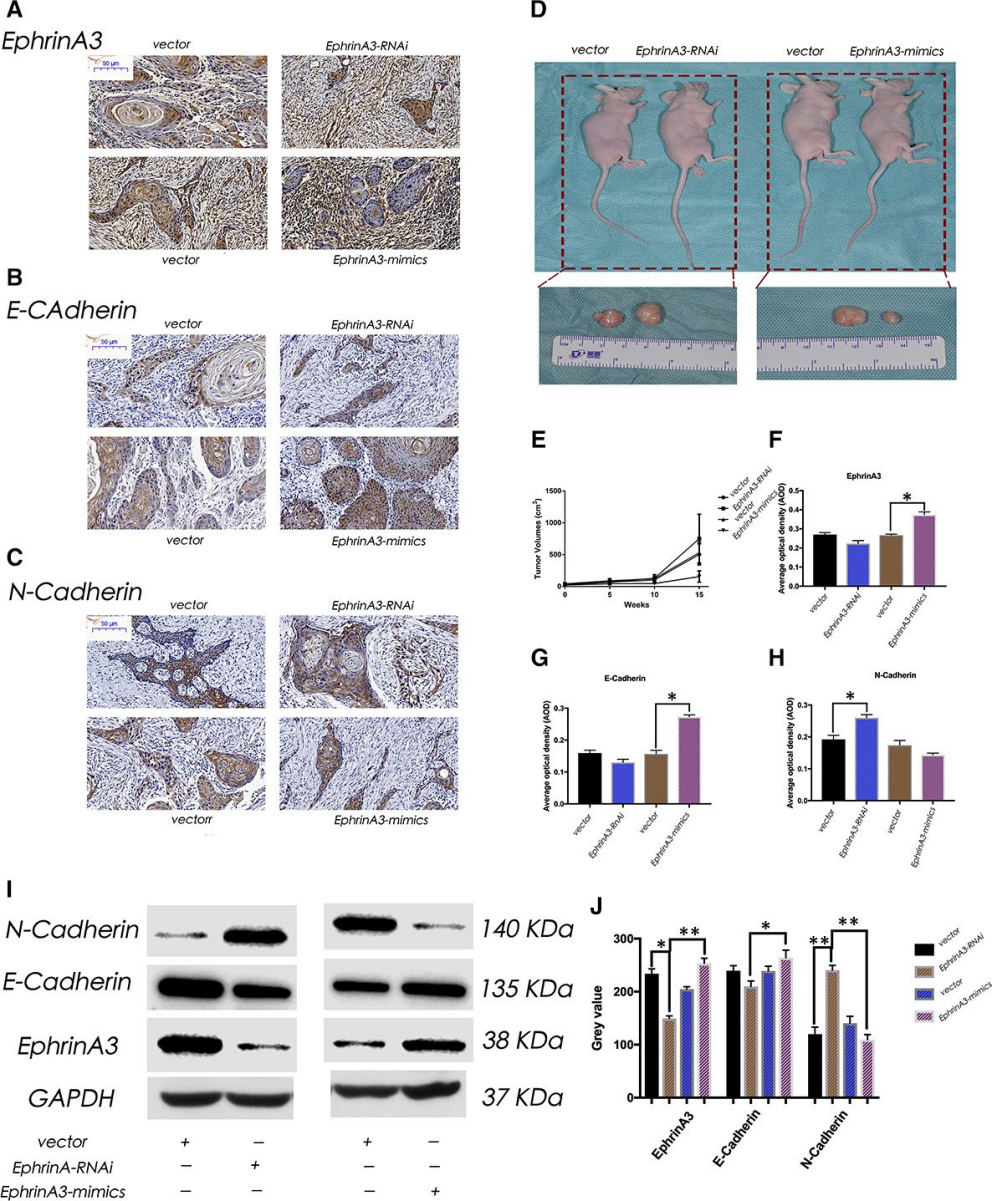
Suppression of EphrinA3 expression promotes tumour proliferation in vivo. (A‐C) shows four groups of tumour specimens were stained with immunohistochemistry. In (F and G), the expression of EphrinA3 and E‐Cadherin in EphrinA3‐RNAi group was different from that in its vector group (*P* < .05)*.* But in (H), the expression of N‐Cadherin in EphrinA3‐RNAi group was different from that in its vector group (*P* < .05)*.* (D, E) showed that the tumour volume in EphrinA3‐RNAi group was greater than that in the EphrinA3‐mimics group (*P* < .05). (I, J) we used the Western blot to analyse the total protein showing that the EphrinA3‐RNAi group was significantly greater than that in the EphrinA3‐mimics group (*P* < .01)

The tumour specimens were stained with immunohistochemistry. The average optical density (AOD) of EphrinA3, E‐cadherin and N‐cadherin were statistically analysed (Figure 4A‐C). The expression of EphrinA3 and E‐cadherin in A3 + group was significantly different from that in the vector group (*P* < .05) (Figure 4F, G). But in the expression of N‐Cadherin in EphrinA3‐RNAi group was statistically different from that in its vector group (*P* < .05) (Figure 6H)*.* The tumour volume in EphrinA3‐RNAi group was greater than that in the EphrinA3‐mimics group, showing statistical differences (*P* < .05) (Figure 4D, H). We also used the Western blot to analyse the total protein showing that the EphrinA3‐RNAi group was significantly greater than that in the EphrinA3‐mimics group, showing statistical differences (*P* < .01; Figure 4I, J). Therefore, knockdown of EphrinA3 expression may promote the proliferation of tumour, while EphrinA3 overexpression can inhibit the proliferation of tumour in vivo.

### 3.6 The miR‐210‐3p targeting the gene EFNA3 can regulate the process of tumour process

**Figure 5:**
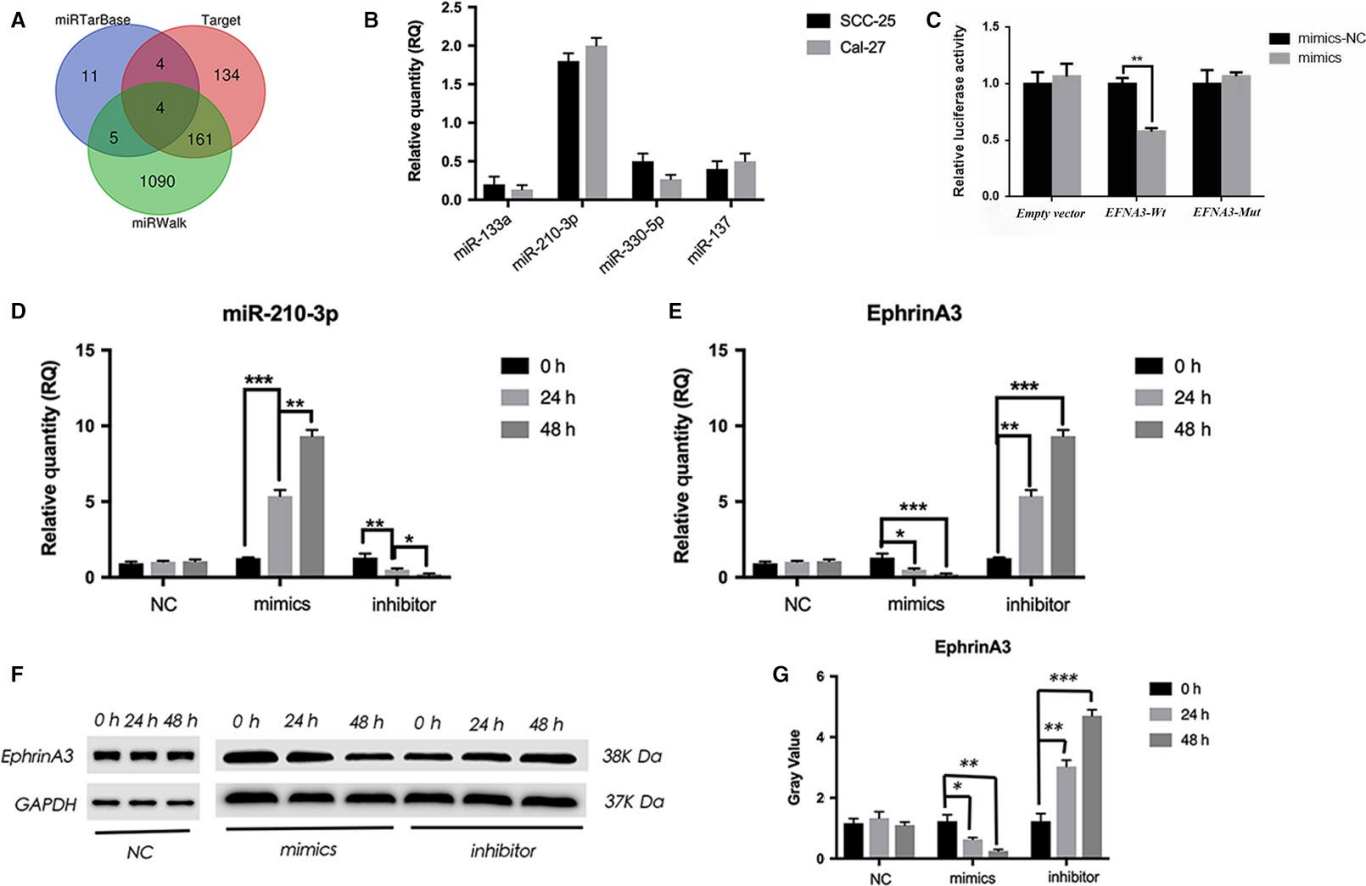
The miR‐210‐3p targeting the gene EFNA3 can regulate the process of tumour process. A, B, showed that four microRNAs with EFNA3 as target genes were predicted by three databases, and the expression of mir‐210‐3p was verified to be high in SCC‐25 and Cal‐27 cell lines. C, D, showed after the transfection of miR‐210 mimics, by real‐time PCR the expression of EphrinA3 decreased with the increase of miR‐210 expression, and the *t* test showed that *P* < .05. E, F, Western blot also showed that with the increase of miR‐210 expression in two cell lines, the EphrinA3 expression decreased. And the decreased expression of miR‐210 could also lead to increased expression of ephrinA3 (*P* < .05)

Using three databases, we predicted four microRNAs targeting the gene EFNA3, and the expression of mir‐210‐3p was verified to be high in SCC‐25 and Cal‐27 cell lines, respectively (Figure 5A, B). In order to further determine that the target gene of miR‐210‐3p is EFNA3, we carried out double luciferase test to confirm our prediction (Figure 5C). After the transfection of miR‐210 mimics in SCC‐25 and Cal‐27 cells, by real‐time PCR the expression of EphrinA3 decreased with the increase of miR‐210 expression, and the *t* test showed that *P* < .05 (Figure 5D, E). Figure F and G showed that Western blot and grey value analysis also showed that with the increase of miR‐210 expression in the SCC‐25 and Cal‐27 cells, the EphrinA3 expression decreased. And the decreased expression of miR‐210 could also lead to increased expression of ephrinA3 (*P* < .05) (Figure 5F, G). So miR‐210‐3p can target the gene EFNA3 to regulate the EMT by the PI3K/AKT signalling pathway.

## 4 DISCUSSION

OSCC is the sixth common cancer worldwide.^24^ Despite advances in cancer diagnosis and treatment, the overall 5‐year survival rate for OSCC remains the lowest among all malignancies and in fact has been < 50% for the last three decades.^2^ Therefore, identification of OSCC biomarkers or effective therapeutic targets is desirable. EMT is a process, wherein an epithelial cell loses its morphology and gains that of a mesenchymal cell. The invasion and metastasis of tumour occurs because of the reduced adhesion between the cancer cells in the course of tumour progression owing to the loss of E‐cadherin expression and increased expression of N‐cadherin.^5^ Walsh et al found that the human epithelium only expressed *EFNA1*, *EFNA3* and *EFNA4*, as well as *EphA1*, *EphA2* and *EphA4*; however, it was negative for the expression of other types of EFNA/EphA genes.^13^ We verified the regulatory effect of miR‐210‐3p expression on *EFNA3* with an aim to identify new targets for the treatment of oral cancer.

The clinical tissue samples of OSCC showed negative correlations between EphrinA3 expression and miR‐210‐3p and E‐cadherin, as well as the pathological grading of tumours.^25^ Knocking‐down EphrinA3 decreased the expression of E‐cadherin and increased the expression of N‐cadherin and p‐AKT, indicating the activation of the PI3K/AKT signalling pathway. EphrinA3 overexpression induced the expression of E‐cadherin but down‐regulated N‐cadherin expression and p‐AKT, suggesting the down‐regulation of the pathway. To explore whether EphrinA3 expression changes cause activation or inhibition of the pathway, we treated with an inhibitor and agonist of the pathway and compared the expression of p‐AKT in EphrinA3 knockdown group, overexpression group and vector group. The treatment with inhibitor resulted in the reduction in the expression of p‐AKT in three groups. However, the addition of agonist increased the expression of p‐AKT in three groups. The level of p‐AKT in EphrinA3 knockdown group was higher than that in vector group, while p‐AKT level was generally lower in EphrinA3 overexpression group than in vector group. Therefore, the addition of agonist (IGF‐1) resulted in an obvious increase in this trend. The down‐regulation effect of EphrinA3 on p‐AKT could be reversed, but this trend was lower than that in vector group and significantly lower than that in EphrinA3 knockdown group. We also conducted functional test of EMT in vitro and in tumour xenograft model. We verified the regulatory effect of miR‐210‐3p on the expression of *EFNA3* to construct a more complete regulatory axis. We found that miR‐210‐3p could reduce the expression of EphrinA3. The down‐regulation of miR‐210‐3p expression could similarly increase the expression of EphrinA3, consistent with the fact that miR‐210‐3p inhibited tumour growth and metastasis.^20^ Taken together, the present study constructed an miR‐210‐3p‐EphrinA3‐PI3K/ATK signalling axis and highlighted that the knockdown of EphrinA3 expression may promote the development of EMT in tumour cells through the activation of PI3K/AKT pathway, thereby promoting tumour growth, invasion and metastasis and drug resistance. Therefore, EphrinA3 may serve as an inhibitor of oral cancer progression and could be used as a target for oral cancer treatment.

The present study has a few limitations. Statistical difference was also observed between the pathological grade I group and pathological grade III group. However, no statistical differences were observed between the pathological grade II group and pathological grade III group. We hypothesized that either the number of cases in pathological grade III group was too small or the difference in EphrinA3 expression between moderately differentiated and poorly differentiated OSCC was not significant. Given the high rate of loss of follow‐up, the relationship between EphrinA3 expression and prognosis of OSCC was not discussed in this study.

## 5 CONCLUSION

The miR‐210‐3p‐EphrinA3‐PI3K/ATK signalling axis plays an important role in EMT of OSCC. Down‐regulation of EphrinA3 expression through this signalling axis may result in the inhibition of the development and metastasis of oral cancer. Therefore, EphrinA3 can be used as a novel target for oral cancer treatment.

## CONFLICTS OF INTEREST

The authors confirm that there are no conflicts of interest.

## AUTHORS' CONTRIBUTIONS

Corresponding author Zhengjun Shang and co‐corresponding author Zhe Shao provided experimental ideas and designs. The author Wang Lin mainly carried on the molecular biology and the cell experiment as well as the article writing. The author Yong Song carried out the experiment of immunohistochemistry and in situ hybridization. The author Hui Wang conducted animal experiments. The author Ke Liu revised the article.

## ETHICAL APPROVAL

Any experimental research on animals should follow internationally recognized guidelines.

## CONSENT FOR PUBLICATION

The author confirms: the work described has not been published before (except in the form of an abstract or as part of a published lecture, review or thesis); that it is not under consideration for publication elsewhere; that its publication has been approved by all co‐authors, if any; that its publication has been approved (tacitly or explicitly) by the responsible authorities at the institution where the work is carried out. The author agrees to publication in the Oral Oncology indicated below and also to publication of the article. The copyright to the English‐language article is transferred to the Journal effective if and when the article is accepted for publication. The author warrants that his/her contribution is original and that he/she has full power to make this grant. The author accepts responsibility for releasing this material on behalf of any and all co‐authors. The copyright transfer covers the exclusive right to reproduce and distribute the article, including reprints, translations, photographic reproductions, microform, electronic form (offline, online) or any other reproductions of similar nature.

## Data Availability

The datasets used or analysed during the current study are available from the corresponding author on reasonable request.
